# Supplementary material for: RedRibbon: A new rank–rank hypergeometric overlap for gene and transcript expression signatures
Source: Life Sci Alliance. 2023 Dec 8;7(2):e202302203. doi: 10.26508/lsa.202302203 (PMC10709657; doi:10.26508/lsa.202302203)
Supplement: Supplementary file 2 [file LSA-2023-02203_Supplemental_Data_2.pdf]

RedRibbon: a rank-rank hypergeometric overlap library

Anthony Piron, Theodora Papadopoulou

2023-11-01

Abstract

High throughput omics technologies have generated a wealth of large protein, gene and transcript datasets that have exacerbated the need for new methods to analyse and compare big datasets. Rank-rank hypergeometric overlap is an important threshold-free method to combine and visualize two ranked lists of P-values or fold-changes, usually from differential gene expression analyses. Here, we introduce a new rank-rank hypergeometric overlap-based method aimed at gene, transcript, exon and protein level capable of taking into account alternative splicing phenomena. It offers the possibility to compare these levels to each other, hitherto unreachable as transcript and protein numbers are an order of magnitude larger than gene numbers.

RedRibbon is a comparative analysis tool of omics differential analyses. It allows to determine the features overlapping between two differential studies. It features high performance, accuracy and simplicity in use. RedRibbon has been optimized to be efficient regardless of the length of the lists given as input. The input data for RedRibbon are two labelled ranked lists of real numbers under comparison. These two lists could contain proteins, gene or transcript ranked by a statistic such as log fold change or direction signed P-value. Optionally, the minimal P-value can be adjusted considering expression level correlation between genes or transcripts.

If you have issues with the `RedRibbon` package, please submit it on the RedRibbon github issue reporting system and we will do our best to solve your problems.

To demonstrate RedRibbon's basic workflow, we will first generate and use a synthetic data set. Next, the tool will be applied on real data sets. We recommend that you read first this simple experimental section to familiarize yourself with the tool before applying it to your real data. Each of the functions or methods described in this R vignette is fully documented. This documentation can be accessed the usual way in R with the question mark prefixing the function name (e.g., `?RedRibbon.data.frame`).

Synthetic data set

The synthetic data set is composed of two labeled lists of numbers. A `data.frame`, called `df`, will contain these lists. This `data.frame` is composed of columns `a` and `b`, the two lists of numbers and `id`, a list of labels. Those columns are mandatory to run RedRibbon rank-rank hyper-geometric overlap. The synthetic lists have one quarter perfectly overlapping at the bottom of the list, one quarter at the top of the list, and the remaining elements are random.

The code to generate those lists in the `data.frame` `df` is

```
set.seed(42)
n <- 1000
n2 <- n %/% 2
n4 <- n %/% 4
a <- (1:n) - n2
b <- a
a[n4:(n4+n2-1)] <- rnorm(n2, sd=100)
b[n4:(n4+n2-1)] <- rnorm(n2, sd=100)
```

```r
df <- data.frame(
    id = paste0("gene", 1:n),
    a = a,
    b = b)
```

The first lines of the `data.frame` (as given by `head(df)`) are

id	a	b
gene1	-499	-499
gene2	-498	-498
gene3	-497	-497
gene4	-496	-496
gene5	-495	-495
gene6	-494	-494

Overlapping

We will now compute an overlap with RedRibbon for this synthetic data set. First, the library is loaded with

```r
library(RedRibbon)
#> Loading required package: scales
#> Loading required package: ggrepel
#> Loading required package: ggplot2
#> Loading required package: data.table
```

Then, we create an S3 RedRibbon object with the `RedRibbon` function,

```r
rr <- RedRibbon(df, enrichment_mode="hyper-two-tailed")
```

We set the parameter `enrichment_mode` to use two-tailed test. This way, we will simultaneously detect enrichment in correlated (up/up and down/down) and anti-correlated (up/down and down/up) genes.

Next, we localize the minimal P-value in the four quadrants with the `quadrants` method on the RedRibbon object,

```r
quad <- quadrants(rr, algorithm="ea", permutation=TRUE, whole=FALSE)
```

We use the evolutionary algorithm (`algorithm="ea"`) to locate the minimal P-value coordinates with the highest accuracy). We also ask the computation of adjusted P-value with the `permutation` option to `TRUE`. `whole=FALSE` splits the overlap map into four quadrants (i.e., down-down, up-up, down-up, up-down).

Plotting the level map

Results can be plotted with the helper function `ggRedRibbon`. The overlap map of the two lists goes respectively in the same direction (both downregulated or both upregulated in the 2 lists). The maximal log P-values and the permutation adjusted P-values are shown for each quadrant. The horizontal and vertical dotted lines split the downregulation and upregulation where the log fold change is zero. The code to produce the map is straightforward,

```r
gg <- ggRedRibbon(rr, quadrants=quad,
          repel.force = 250) +
          coord_fixed(ratio = 1, clip = "off")
gg
```

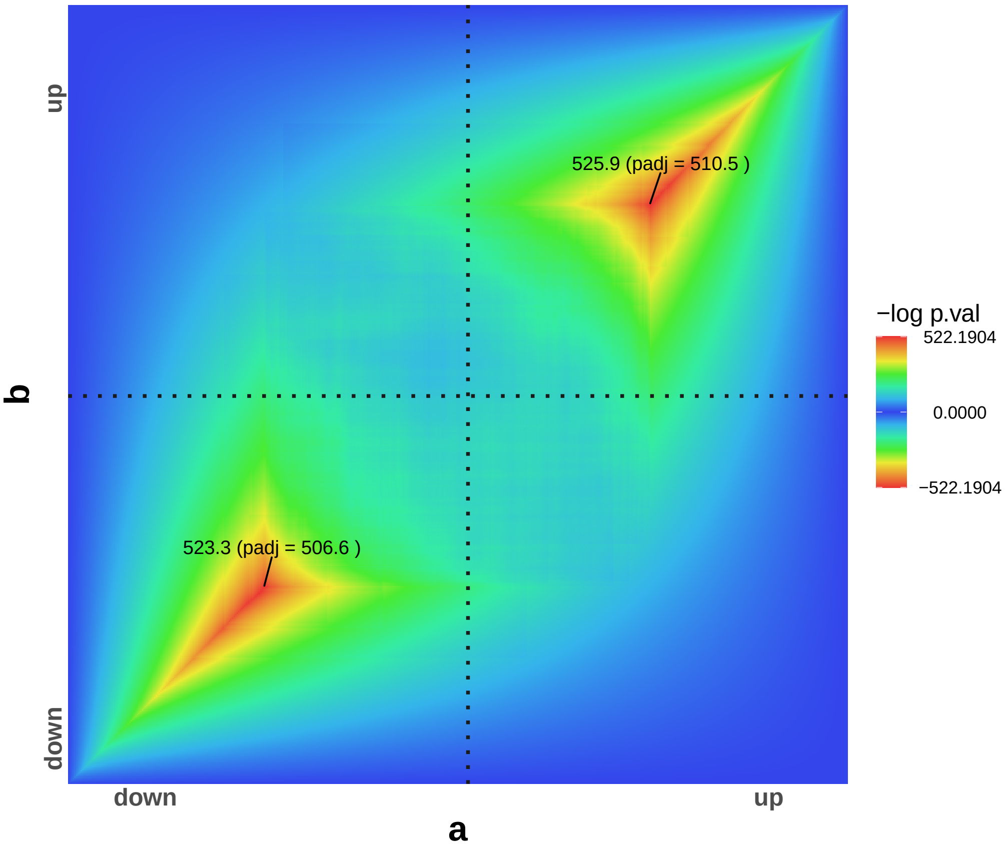

In the overlap map, the hypergeometric P-value are signed negatively for depletion (anti-correlation) and positive for enrichment (correlation).

The `gg` variable contains a standard ggplot2 object,

```
class(gg)
#> [1] "gg"     "ggplot"
```

Hence, this object can be manipulated as any ggplot object. For example, in this vignette we used `+ coord_fixed(ratio = 1)` to obtain a fixed scale of coordinates (see `?ggplot2::coord_fixed`).

Enrichment statistics

The lists of overlapping genes are returned by the `quadrants` method. The list of genes enriched in the down-down direction is given by `df[quad$downdown$positions,]`

id	a	b
gene1	-499	-499
gene2	-498	-498
gene3	-497	-497
gene4	-496	-496
gene5	-495	-495
gene6	-494	-494

`quad$downdown$positions` is a vector of indices in the `df` `data.frame`. The other quadrants indices are given in `quad$upup$positions`, `quad$downup$positions`, and `quad$updown$positions`.

You may also easily access all the statistics generated by the analysis:

```r
names(quad$downdown)
#> [1] "pvalue"     "log_pvalue" "direction"  "count"      "positions"
#> [6] "i"          "j"          "padj"       "log_padj"
```

```r
quad$downdown[c("i", "j", "pvalue", "log_pvalue", "count", "direction")]
#> $i
#> [1] 252
#>
#> $j
#> [1] 253
#>
#> $pvalue
#> [1] 5.386098e-228
#>
#> $log_pvalue
#> [1] 523.3056
#>
#> $count
#> [1] 249
#>
#> $direction
#> [1] 1
```

`(i , j)` is the minimal P-value coordinate on the enrichment map, `pvalue` and `log_pvalue` are the minimal P-value, `direction` is the direction of the enrichment. `count` is the number of genes overlapping.

Compatibility mode

This mode only exists for compatibility with RRHO. Please use the new interface shown above when possible.

```r
compat <- RRHO(df[,c("id", "a")], df[,c("id", "b")], plots=TRUE, stepsize=n %/% 10)
#> Warning in RRHO(df[, c("id", "a")], df[, c("id", "b")], plots = TRUE, stepsize
#> = n%/%10): This function only exists for compatibility. Please use instead the
#> RedRibbon() call. See Vignette("RedRibbon") for details.
compat$gg
```

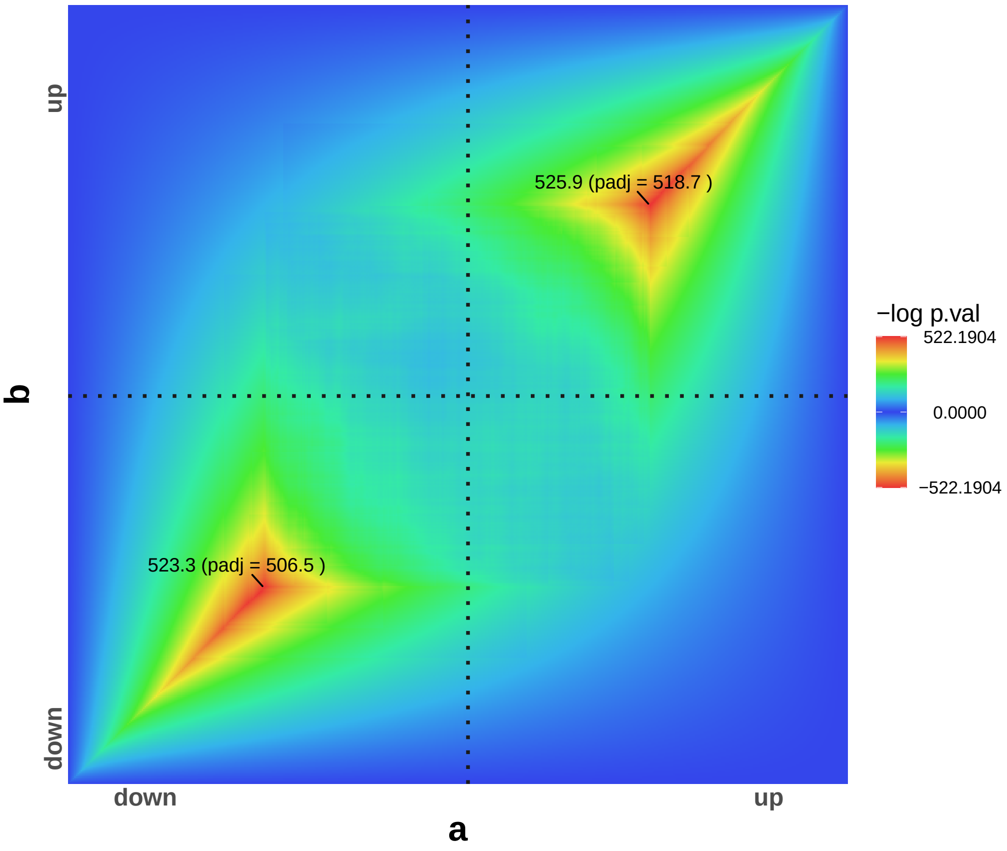


```
compat$hypermat
```

6.21	1.60	0.91	0.51	0.22	0.00	0.00	0.00	0.00	0.00
1.60	323.41	186.97	134.38	100.17	74.72	54.42	37.53	23.08	10.43
0.91	186.97	475.05	302.06	216.51	157.72	112.69	81.00	49.81	22.84
0.51	134.38	295.53	301.82	220.29	172.50	133.25	102.82	75.94	37.03
0.22	100.17	210.82	206.99	161.51	130.69	135.74	132.97	112.12	53.61
0.00	74.72	157.72	157.20	139.90	115.81	128.18	168.74	156.78	73.54
0.00	54.42	118.00	139.64	143.47	137.29	179.23	240.46	215.13	98.55
0.00	37.53	81.00	121.90	142.67	171.89	229.69	330.92	293.58	132.19
0.00	23.08	49.81	80.73	117.42	162.59	221.52	307.34	462.53	183.96
0.00	10.43	22.84	37.03	53.61	73.54	98.55	132.19	183.96	319.02

For the description of these field and parameters, we refer the reader to the `RRHO` package documentation.

Using and computing correlation between genes

To demonstrate how we use the correlation of expression between genes, we will generate an artificial expression matrix of `n.genes x n.samples` associated to a fold change between the 2 conditions (e.g., treated vs non-treated samples). The first five genes are perfectly correlated.

```
gen.expression.matrix <- function (n.genes, n.samples, fc=NULL, r = NULL)
{
    n.treated <- n.samples %/% 2
    n.non.treated <- n.samples - n.treated
```

```
    mat.non.treated <- matrix( rnorm(n.genes * n.non.treated, sd=10), nrow = n.genes)
    mat.fc <- if (is.null(fc)) 0 else mat.non.treated * fc
    mat.treated <- matrix(mat.fc + rnorm(n.genes * n.non.treated, sd=10) , nrow = n.genes)
    mat <- cbind(
                 mat.non.treated,
                 mat.treated)
    if ( ! is.null(r) )
    {
           for (i in 2:(length(r)+1) )
         {
            mat[i, ] <- mat[1,] * r[i-1] + (1-abs(r[i-1])) * mat[i, ]
         }
    }
    mat
}

n <- 50
n.frac <- 5
fc_a <- c(rep(-2, n.frac), runif(n-n.frac, -2, 2))
fc_b <- c(rep(-2, n.frac), runif(n-n.frac, -2, 2))
df <- data.frame(a=fc_a, b=fc_b)

mat_b <- gen.expression.matrix(n, 6, fc=fc_b, r=c(1, 1, 1, 1))

head(mat_b)
#>          [,1]      [,2]      [,3]      [,4]       [,5]     [,6]
#> [1,] 3.987343 26.024689 -4.134666 -3.566446 -51.725370 15.19353
#> [2,] 3.987343 26.024689 -4.134666 -3.566446 -51.725370 15.19353
#> [3,] 3.987343 26.024689 -4.134666 -3.566446 -51.725370 15.19353
#> [4,] 3.987343 26.024689 -4.134666 -3.566446 -51.725370 15.19353
#> [5,] 3.987343 26.024689 -4.134666 -3.566446 -51.725370 15.19353
#> [6,] 4.311339  6.004302 26.337096 13.933081  -4.466707 30.20824
```

We can then compute the correlation,

```
correlation <- rrho_expression_prediction(mat_b)
c.obj <- newFC(correlation$index, correlation$beta[,2])
c.obj
#> $deps
#>  [1]  3  3 -1  3  3 -1 -1 -1 -1 -1 -1 -1 -1 -1 -1 -1 -1 -1 -1 -1 -1 -1 -1 -1 -1
#> [26] -1 -1 -1 -1 -1 -1 -1 -1 -1 -1 -1 -1 -1 -1 -1 -1 -1 -1 -1 -1 -1 -1 -1 -1 -1
#>
#> $beta
#>  [1] 1 1 0 1 1 0 0 0 0 0 0 0 0 0 0 0 0 0 0 0 0 0 0 0 0 0 0 0 0 0 0 0 0 0 0 0 0 0
#> [39] 0 0 0 0 0 0 0 0 0 0 0 0
#>
#> attr(,"class")
#> [1] "fc"
```

And input the computed correlation in `RedRibbon`

```
rr <- RedRibbon(df, enrichment_mode="hyper-two-tailed", correlation=c.obj)
quad <- quadrants(rr, algorithm="ea", permutation=TRUE, whole=FALSE)
gg <- ggRedRibbon(rr, quadrants=quad,
          repel.force = 250) +
```

```
   coord_fixed(ratio = 1, clip = "off")
gg
```

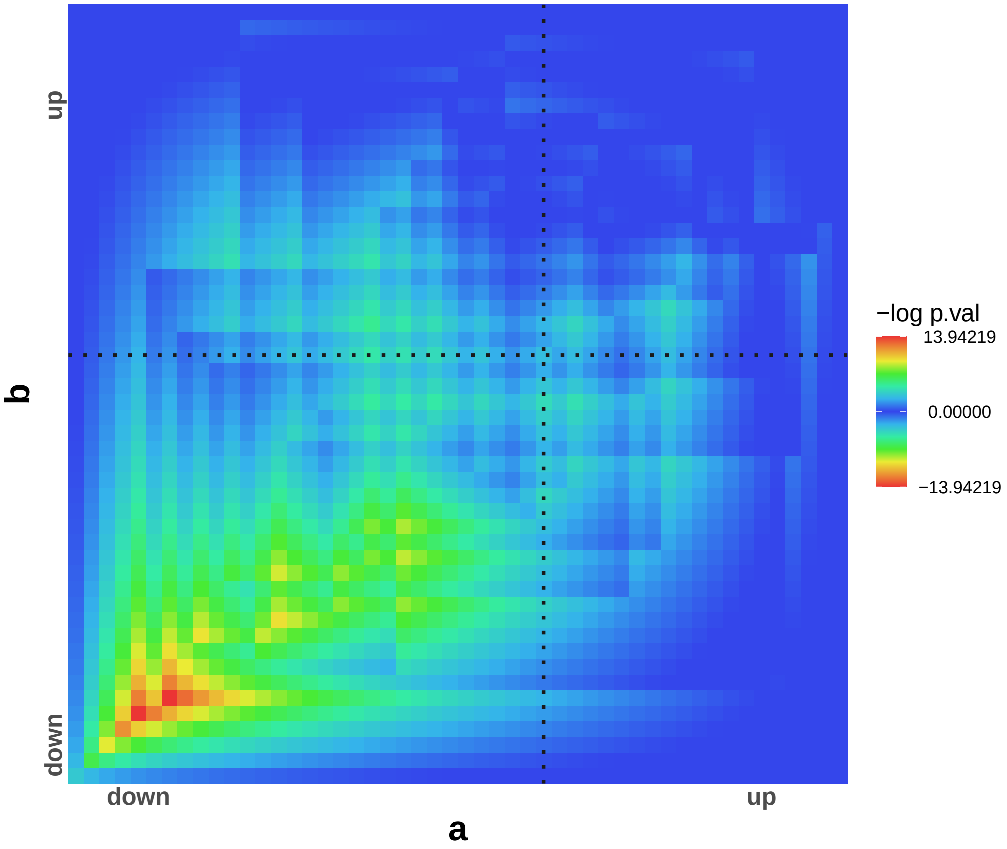

Considering gene correlations, the adjusted P-value of the overlap is not significant. Hence, no minimal coordinate is shown on the map.

Analysis with real data sets

To demonstrate the versality and potential of `RedRibbon`, we will apply it to diverse omic data sets. The method is agnostic to the underlying technology. Here, this robustness will be illustrated with RNA-Seq transcriptome (quantified at gene and transcript level), micro-array, and proteome.

Overlap of transcriptomic analyses

To demonstrate the capabilities of `RedRibbon`, we will use data from GSE159984 GEO submission. This data set has been analyzed in depth in the RedRibbon main manuscript. The data contains the output from two `DESeq2` differential expression analyses. In this case, columns `a` and `b` contain log2FoldChange values corresponding to type 2 diabetes islet signatures (`T2DvsCTL`) and human islets treated with palmitate and glucose signatures (`D8PGsCTL`).

First, we load the data set and appropriately name the mandatory columns (`id`, `a` and `b`),

```
data("T2D-D8PG", package = "RedRibbon")

df.real <- merge(T2DvsCTL[, c("ensembl_id", "log2FoldChange")],
                 D8PGvsCTL[, c("ensembl_id", "log2FoldChange")],
         by = "ensembl_id")
```

```r
colnames(df.real) <- c("id", "a", "b")
head(df.real) %>%
  kbl() %>%
  kable_styling(latex_options = "HOLD_position")
```

id	a	b
ENSG00000000003	-0.0782417	-0.5622385
ENSG00000000419	-0.0272121	-0.0749227
ENSG00000000457	0.0250109	-0.1753647
ENSG00000000460	-0.0015721	0.0168077
ENSG00000000938	0.5226592	-0.4279560
ENSG00000000971	-0.2215815	-1.1212551

Then, we run `RedRibbon`,

```r
rr <- RedRibbon(df.real, enrichment_mode="hyper-two-tailed")
quad <- quadrants(rr, algorithm="ea", permutation=TRUE, whole=FALSE)
```

`RedRibbon` function constructs the object which will be used by `quadrants` to compute the overlap in the four regulation quadrants. All the steps performed by `RedRibbon` and `quadrants` are described in the RedRibbon manuscript.

Then, `ggRedRibbon` is used to plot the overlap map.

```r
gg <- ggRedRibbon(rr, quadrants=quad,
              labels = c("T2D vs CTL", "D8PG vs CTL"),
          repel.force = 250) +
   coord_fixed(ratio = 1, clip = "off")
gg
```

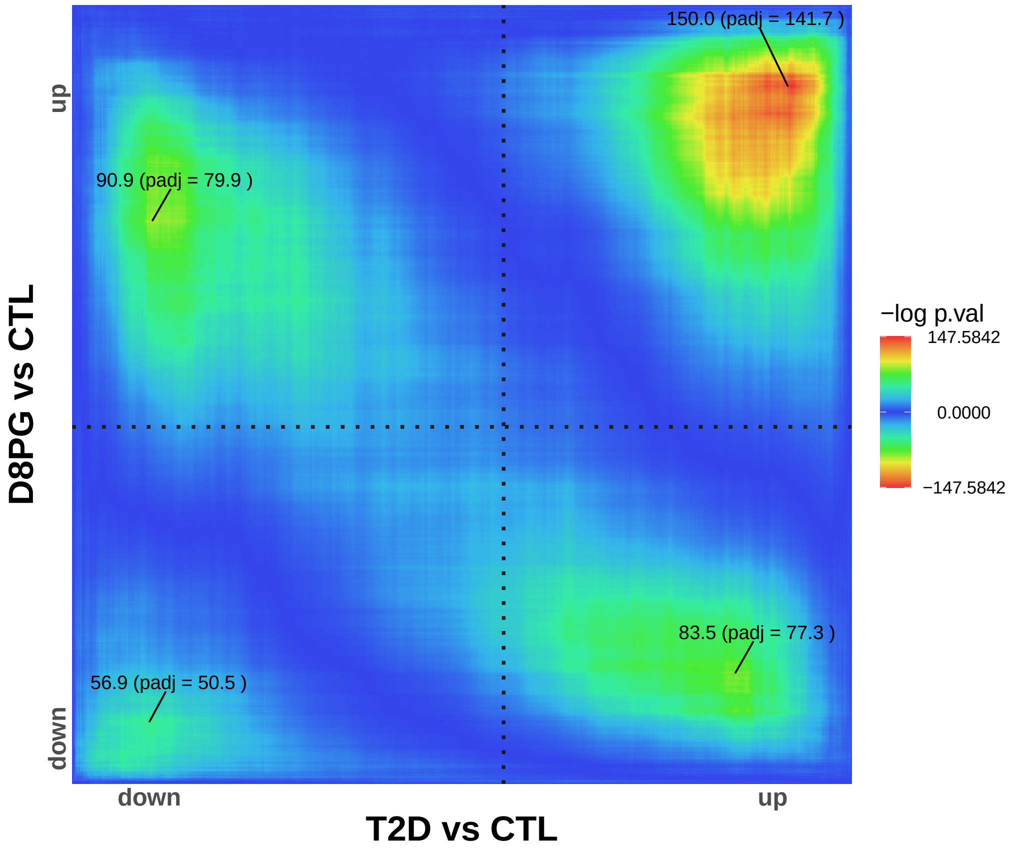

gg is a standard ggplot2 object. All the ggplot2 methods can be applied on it. Here, the coord_fixed ggplot2 method is called to guarantee a fixed square ratio.

Long read analysis compared to short read analysis

RedRibbon is capable of handling both long and short read RNA-Seq differential analyses. To illustrate this, we will compare the long (GSE167223) and short read (GSE137136) signatures of pancreatic beta cells exposed to cytokines. This data set has also been analyzed in the RedRibbon main manuscript.

This dataset is included in the R package. The gene level analyses are loaded with

```
data("CYT-Short-Long", package = "RedRibbon")
df.genes <- merge(CYTvsCTL_short_genes[, c("ensg", "log2FoldChange")],
                  CYTvsCTL_long_genes[, c("ensg", "log2FoldChange")],
                  by = "ensg")
colnames(df.genes) <- c("id", "a", "b")

head(df.genes) %>%
  kbl() %>%
  kable_styling(latex_options = "HOLD_position")
```

id	a	b
ENSG00000000419	-0.0216161	-0.0964418
ENSG00000000457	0.7003209	0.9418924
ENSG00000000460	-1.1426132	-0.0032452
ENSG00000001036	0.0373025	0.2422237
ENSG00000001084	-0.5730671	-0.2861927
ENSG00000001167	-0.3753396	0.5775642

And then, we run RedRibbon analysis over the `df.genes` merged table,

```
sh_vs_l_genes <- RedRibbon(df.genes, enrichment_mode="hyper-two-tailed")
quad <- quadrants(sh_vs_l_genes, algorithm="ea", permutation=TRUE, whole=FALSE)
gg <- ggRedRibbon(sh_vs_l_genes, quadrants=quad,
                  labels = c("short-reads CYT vs CTL (gene level)", "long-reads CYT vs CTL (gene level)
                  repel.force = 250) +
    coord_fixed(ratio = 1, clip = "off")
gg
```

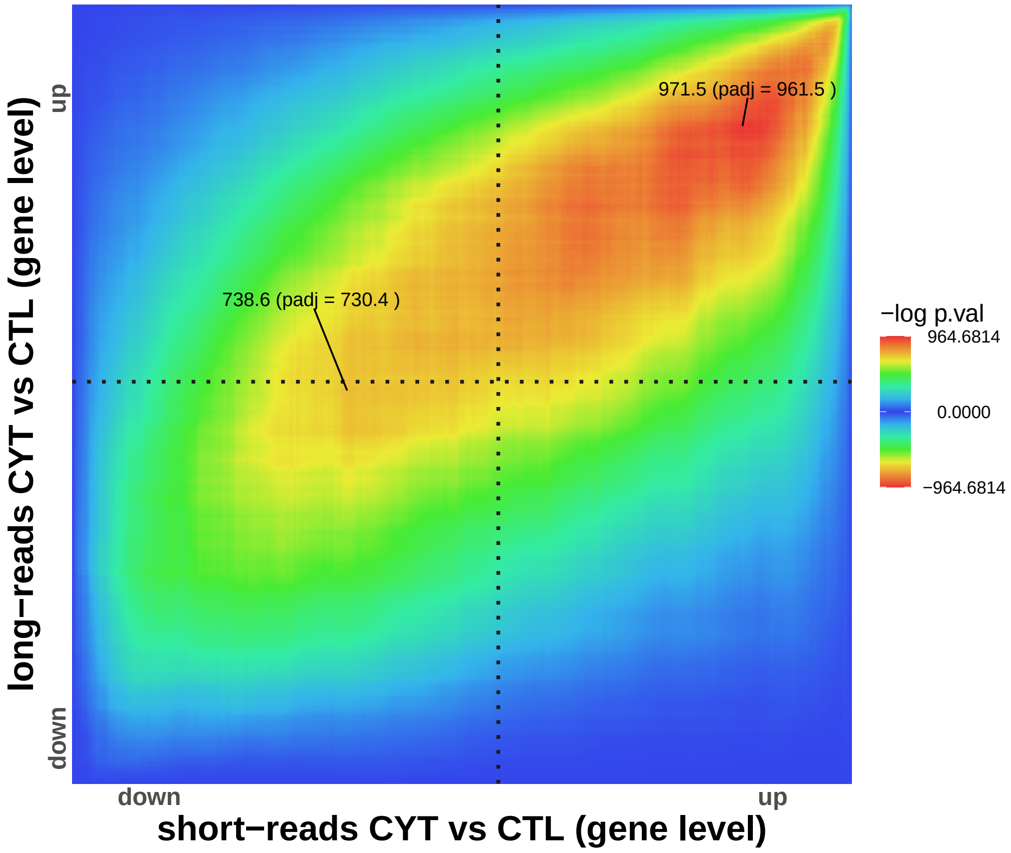

At transcript level, we have

```
data("CYT-Short-Long-tx", package = "RedRibbon")
df.tx <- merge(CYTvsCTL_short_tx[, c("Row.names", "log2FoldChange")],
                  CYTvsCTL_long_tx[, c("Row.names", "log2FoldChange")],
                 by = "Row.names")
colnames(df.tx) <- c("id", "a", "b")

head(df.tx) %>%
  kbl() %>%
  kable_styling(latex_options = "HOLD_position")
```

id	a	b
ENST00000000233	-0.2739232	0.0203169
ENST00000000412	0.0908180	0.1113825
ENST00000000442	0.2294083	2.2626586
ENST00000001008	0.1581945	0.0847822
ENST00000002125	0.0041481	0.5701458
ENST00000002165	0.0332171	0.2407866

```
sh_vs_l_tx <- RedRibbon(df.tx, enrichment_mode="hyper-two-tailed")
quad <- quadrants(sh_vs_l_tx, algorithm="ea", permutation=TRUE, whole=FALSE)
gg <- ggRedRibbon(sh_vs_l_tx, quadrants=quad,
                  labels = c("short-reads CYT vs CTL (transcript level)",
                             "long-reads CYT vs CTL (transcript level)"),
                  repel.force = 250) +
    coord_fixed(ratio = 1, clip = "off")
gg
```

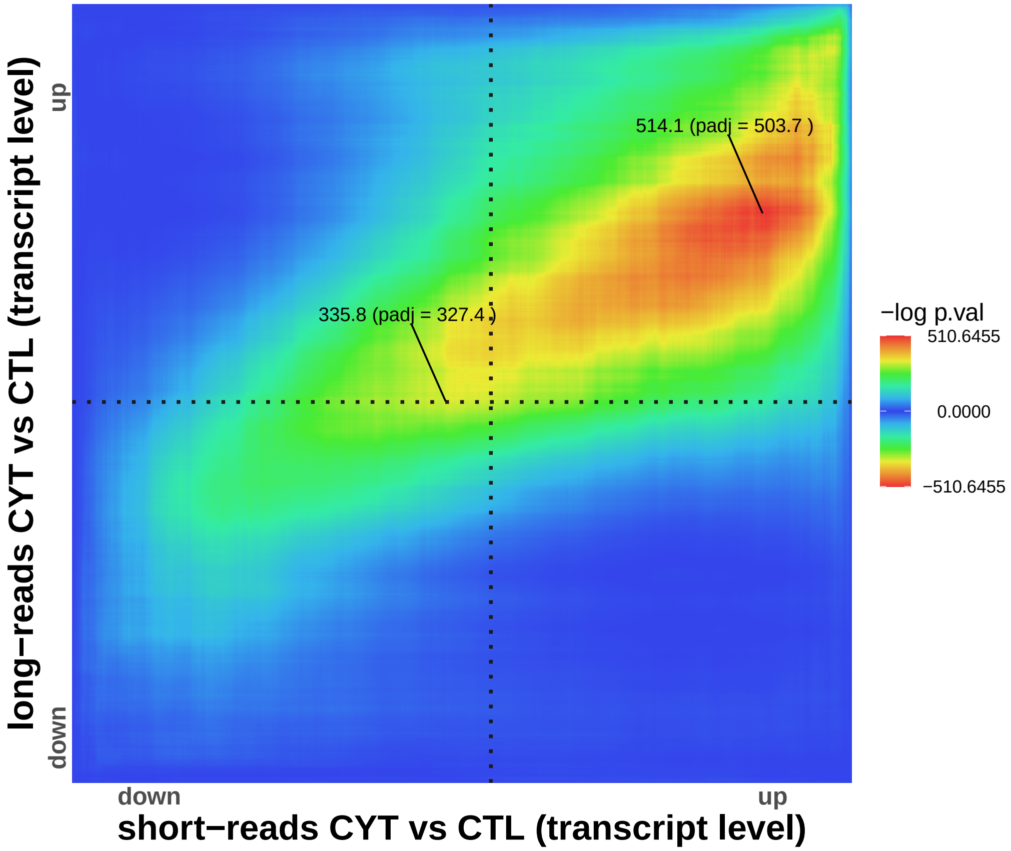

Remark: The hypergeometric P-values on this figure can be slightly different than the same figure in the RedRibbon manuscript, the evolutionary algorithm being non-deterministic. In this specific case, it is caused by diffuse signal around the minimal P-value coordinate. Hence, multiple coordinates have nearly identical P-value. The coordinate being close, this non-determinism have nearly no consequence on the returned overlap.

Proteomic analysis vs transcriptomic analysis

The method allows to compare diverse omic types. One of the interesting overlap comparisons is between transcriptome and proteome for the same cells and treatments as it allows to see if changes in transcriptome recapitulate in the proteome. To illustrate this point, we re-analyzed the data set from Lytrivi et al., 2020 for INS-1E cells treated with palmitate.

The data set is loaded with

```
data("PAL_MAvsProt", package = "RedRibbon")
head(PAL_MAvsProt) %>%
  kbl() %>%
  kable_styling(latex_options = "HOLD_position")
```

id	a	b
A1BG	-0.1731636	1.01
A1CF	-0.1131687	-1.02
AAAS	0.1052605	-1.51
AACS	-0.1109316	-1.51
AAK1	-0.0650720	-1.00
AAK1	-0.0650720	-1.01

The `RedRibbon` overlap is done the usual way,

```
rr <- RedRibbon(PAL_MAvsProt, enrichment_mode = "hyper-two-tailed")
quad <- quadrants(rr, algorithm="ea", permutation=TRUE, whole=FALSE)
gg <- ggRedRibbon(rr, quadrants=quad,
              labels = c("Proteome Palmitate 24h", "Microarray Palmitate 14h"),
          repel.force = 250) +
   coord_fixed(ratio = 1, clip = "off")
gg
```

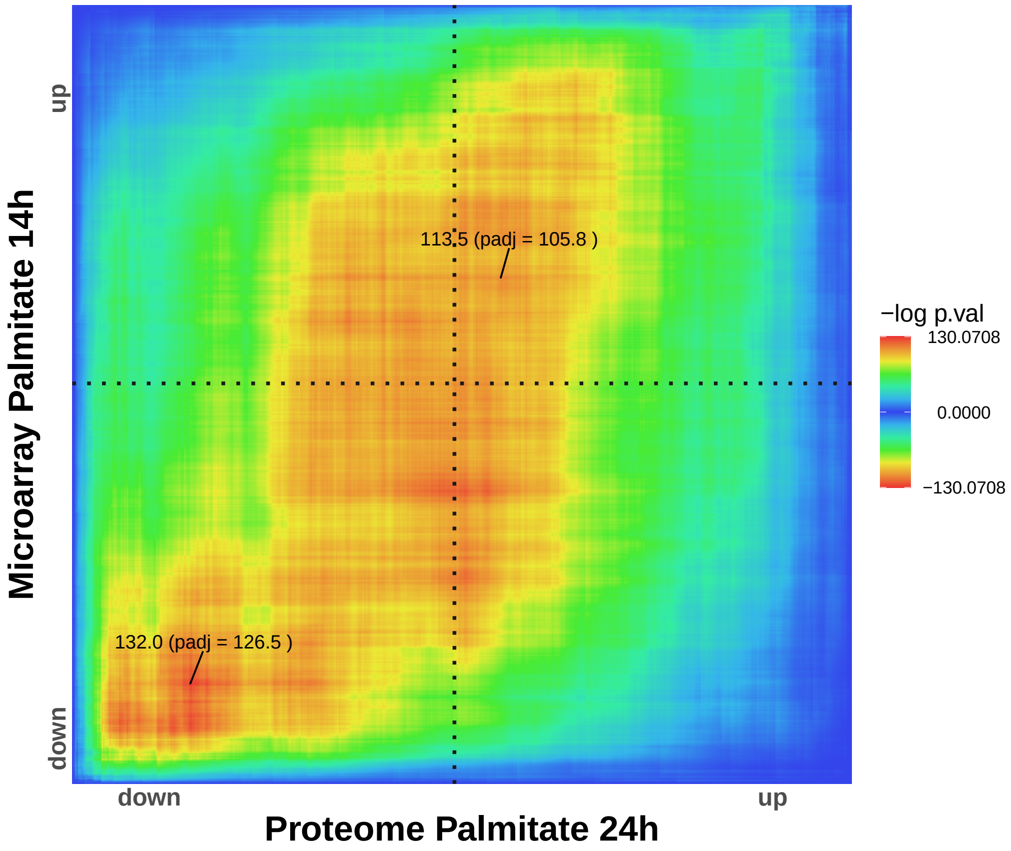

A nice overlap is observed on the major diagonal showing that most changes observed at transcript level recapitulate at protein level. Worth of note, in Lytrivi et al., 2020 with the original R RRHO package (Plaisier et al., 2010), we were unable to pinpoint the minimal hyper-geometric *P*-value coordinate. Here, the improved accuracy of RedRibbon allows the detection of up-up quadrant overlap.

sessionInfo()

```
sessionInfo()
#> R version 4.3.1 (2023-06-16)
#> Platform: x86_64-pc-linux-gnu (64-bit)
#> Running under: Debian GNU/Linux trixie/sid
#>
#> Matrix products: default
#> BLAS:   /usr/lib/x86_64-linux-gnu/openblas-pthread/libblas.so.3
#> LAPACK: /usr/lib/x86_64-linux-gnu/openblas-pthread/libopenblasp-r0.3.24.so;  LAPACK version 3.11.0
#>
#> locale:
#>  [1] LC_CTYPE=en_US.UTF-8       LC_NUMERIC=C
#>  [3] LC_TIME=en_US.UTF-8        LC_COLLATE=en_US.UTF-8
#>  [5] LC_MONETARY=en_US.UTF-8    LC_MESSAGES=en_US.UTF-8
#>  [7] LC_PAPER=en_US.UTF-8       LC_NAME=C
#>  [9] LC_ADDRESS=C               LC_TELEPHONE=C
#> [11] LC_MEASUREMENT=en_US.UTF-8 LC_IDENTIFICATION=C
#>
#> time zone: Europe/London
#> tzcode source: system (glibc)
```

```
#>
#> attached base packages:
#> [1] stats     graphics  grDevices utils     datasets  methods   base
#>
#> other attached packages:
#> [1] RedRibbon_0.4-1   data.table_1.14.6 ggrepel_0.9.3     ggplot2_3.4.1
#> [5] scales_1.2.1      kableExtra_1.3.4
#>
#> loaded via a namespace (and not attached):
#>  [1] gtable_0.3.1      dplyr_1.0.10      compiler_4.3.1    Rcpp_1.0.10
#>  [5] webshot_0.5.4     tidyselect_1.2.0  xml2_1.3.5        stringr_1.5.0
#>  [9] assertthat_0.2.1  systemfonts_1.0.4 yaml_2.3.6        fastmap_1.1.1
#> [13] R6_2.5.1          generics_0.1.3    knitr_1.41        tibble_3.1.8
#> [17] munsell_0.5.0     DBI_1.1.3         svglite_2.1.1     pillar_1.8.1
#> [21] rlang_1.0.6       utf8_1.2.3        stringi_1.7.12    xfun_0.36
#> [25] viridisLite_0.4.1 cli_3.6.0         withr_2.5.0       magrittr_2.0.3
#> [29] digest_0.6.31     rvest_1.0.3       grid_4.3.1        rstudioapi_0.14
#> [33] lifecycle_1.0.3   vctrs_0.5.2       evaluate_0.19     glue_1.6.2
#> [37] farver_2.1.1      fansi_1.0.4       colorspace_2.1-0  rmarkdown_2.25
#> [41] httr_1.4.4        tools_4.3.1       pkgconfig_2.0.3   htmltools_0.5.4
```
